# Impaired Self-Referential Cognitive Processing in Bipolar Disorder: A Functional Connectivity Analysis

**DOI:** 10.3389/fnagi.2022.754600

**Published:** 2022-02-07

**Authors:** Jian Zhang, Tiantian Liu, Zhongyan Shi, Shuping Tan, Dingjie Suo, Chunyang Dai, Li Wang, Jinglong Wu, Shintaro Funahashi, Miaomiao Liu

**Affiliations:** ^1^School of Mechatronical Engineering, Beijing Institute of Technology, Beijing, China; ^2^School of Life Sciences, Beijing Institute of Technology, Beijing, China; ^3^Center for Psychiatric Research, Beijing Huilongguan Hospital, Beijing, China; ^4^Cognitive Neuroscience Laboratory, Graduate School of Natural Science and Technology, Okayama University, Okayama, Japan; ^5^Advanced Research Institute of Multidisciplinary Sciences, Beijing Institute of Technology, Beijing, China; ^6^School of Psychology, Shenzhen University, Shenzhen, China

**Keywords:** bipolar disorder, social cognitive processing, phase lag index, functional connectivity, machine learning classification

## Abstract

Patients with bipolar disorder have deficits in self-referenced information. The brain functional connectivity during social cognitive processing in bipolar disorder is unclear. Electroencephalogram (EEG) was recorded in 23 patients with bipolar disorder and 19 healthy comparison subjects. We analyzed the time-frequency distribution of EEG power for each electrode associated with self, other, and font reflection conditions and used the phase lag index to characterize the functional connectivity between electrode pairs for 4 frequency bands. Then, the network properties were assessed by graph theoretic analysis. The results showed that bipolar disorder induced a weaker response power and phase lag index values over the whole brain in both self and other reflection conditions. Moreover, the characteristic path length was increased in patients during self-reflection processing, whereas the global efficiency and the node degree were decreased. In addition, when discriminating patients from normal controls, we found that the classification accuracy was high. These results suggest that patients have impeded integration of attention, memory, and other resources of the whole brain, resulting in a deficit of efficiency and ability in self-referential processing.

## Introduction

Bipolar disorder (BD) is a common psychiatric illness that is characterized by extreme changes in mood between mania and depression. Compared with depressive disorder, BD has a more complex clinical presentation and a course, a more difficult treatment, a poorer prognosis, and a higher risk of suicide (Mathers et al., 2006; Crump et al., 2013; Librenza-Garcia et al., 2017). Neurocognitive deficits have been shown in patients with BD, such as attention and memory deficits and damage in language and motor functions (Dols et al., 2018).

In recent years, many studies have identified that patients with BD have abnormalities in self-referential cognition, including lack of self-confidence (Jones et al., 2005), excessive introspection (Thomas et al., 2007), and dysfunctional self-attitudes (Scott and Pope, 2003). Self-referential processing refers to conscious decision-making on matters related to self (van der Meer et al., 2010). Self-referential processing is an important component of self and social cognitio (Harvey et al., 2011; Shad et al., 2011). A large amount of clinical evidence also shows that the cognitive impairment of schizophrenia and BD is closely related to the self-referential processing of patients (Lombardo et al., 2010; Carrion et al., 2011; Gilleen et al., 2011). The studies found that patients with schizophrenia had abnormal activation in the anterior and posterior cortical midline structures in response to self-reflection stimuli (Blackwood et al., 2004; Holt et al., 2011). For bipolar episodes, van der Gucht et al. (2009) suggested negative cognition during depression, and Zhao (Zhao et al., 2016) found that self- and other kinds of referential processing were defective in patients with bipolar.

With further exploration of cognitive deficits for BD, substantial studies suggest that the brain dysfunction of BD is caused by a defect in the connections between brain regions, such as the ventral-limbic network connectivity reduced and enhancing emotional responses, fronto-insular and fronto-cingulate connectivity reduced in selective attention (Calhoun et al., 2011; Frangou, 2012; Houenou et al., 2012). Alterations in prefrontal-limbic coupling of patients with BD were found by magnetic resonance imaging (MRI; Strakowski et al., 2012; Anticevic et al., 2013). Although MRI has the characteristics of high spatial resolution; it is based on blood oxygenation level dependent (BOLD). It simulates the hemodynamic processes of the brain and only indirectly reflects the neuronal activity (Salvador et al., 2005). In contrast, the synchronized electrical activity of large populations of neurons is measured using electroencephalogram (EEG) with excellent temporal resolution (Kim et al., 2013). EEG is the most suitable technique for studying brain function on the time scale of nerves. Moreover, the EEG can evaluate instantaneous interactions (functional connectivity). The synchronization likelihood with EEG was used to measure the functional connectivity of the brain in BD (Kim et al., 2013). The differences from EEG coherence between BD and normal control (NC) in the resting state were studied (Kam et al., 2013; Handayani et al., 2017). The self-disorder has become a core feature of BD (Zhao et al., 2016); however, evidence for real brain connection abnormalities in BD is still limited.

In order to further explore the defects of brain functional connectivity in the process of self-cognition for BD, The EEG techniques are excellent for evaluating the time course of cognitive processing, given ERP’s high temporal resolution compared with fMRI. The former study readily obtained functional connectivity in the BD brain by estimating statistical interdependencies between the EEG signals recorded over different brain regions (Kam et al., 2013; Handayani et al., 2017). However, using the phase lag index (PLI) to build functional connections in the brain enables the removal and attenuation of the synchronization that occurs at or near the zero phase difference (Doesburg et al., 2013; Wang et al., 2017; Zhang et al., 2018). Then, the effect of spurious synchronization, which originates from common sources and volume conduction, is reduced. PLI is a synchronization measure and reflects the degree of phase change between tests within a given frequency range (Stam et al., 2007; van Diessen et al., 2013). Using PLI, some studies found degradation of the functional connection of the brain in Alzheimer’s disease (Stam et al., 2007), schizophrenia (Yan et al., 2017), and old age (Wang et al., 2017). Moreover, abnormal brain network topological metrics provide a new perspective for neurological disease diagnosis. This approach has been used in various brain diseases, such as Alzheimer’s disease (Crump et al., 2013), schizophrenia (Jones et al., 2005), and BD (Kim et al., 2013).

In general, we expected that the self-reference impairment of BD might be caused by the poor functional connectivity in the whole brain. So, the purpose of this study is to verify whether aberrant connectivity can lead to self-reference cognitive impairment in patients with BD. To deepen the comprehensive understanding of the characteristics of self-reference processing in patients with BD and promote the corresponding intervention work, we discuss the brain network connection mechanism of self-reference processing defects in the present study. We used the self-referential memory (SRM) task in which participants engaged in a personality trait adjective evaluation test task. Then, the brain functional connectivity network was built from the time series of EEG data to calculate the statistical features of the brain functional network and to study the connections between brain regions. Therefore, by discussing the brain connection characteristics of self-reference processing in BD, we provide a new perspective for treatment and rehabilitation from a neurotransmission perspective.

## Materials and Methods

### Subjects

The participants included 23 patients with bipolar disorder (BD) I disorder (12 males; age, 34.3 ± 12 years) and 19 healthy comparison subjects (NC: 11 males; age, 32.6 ± 8.2 years). The number of subjects required for the experiment was determined by *a priori* power analysis using G*Power based on the assumption of a general linear model, detection of medium effect sizes = 0.25, a type I-error probability of α = 0.05 and an analytical power of (1-β) = 0.95 (Faul et al., 2007). The patients were recruited from the inpatient department at the Beijing HuiLongGuan Hospital. The diagnostic criteria of BD were applied according to the Diagnostic and Statistical Manual of Mental Disorders-IV (American Psychiatric Association, 2000), and the patients with BD had no history of significant neurological or medical problems. The Young Mania Rating Scale (YMRS; Hamilton, 1967), the 21-item Hamilton Depression scale (HAMD; Young et al., 1978), and the Clinical Global Impression-Severity scale (CGI-S; Guy, 1976) were used in the clinical assessment. The healthy subjects were recruited through postings from the surrounding community or were employees of the Beijing HuiLongGuan Hospital. The healthy subjects had no past psychiatric history and no family history of any mental health-related problems. The study was approved by The Research Ethics Committee of the Beijing HuiLongGuan Hospital and carried out according to the Helsinki Declaration of 1975. Prior to the experiment, all the subjects had signed an informed consent form and were paid for their participation. There was no significant difference between the two groups regarding age, education, or gender. The socio-demographic and clinical data are provided in the supplementary material (Supplementary Table 1).

### Materials and Procedure

We used the self-referential memory (SRM) paradigm previously described by the research team (Zhao et al., 2014, 2016). The SRM task involved an encoding phase and a recognition phase. In the experiment, there were 310 personality trait adjectives (2.39° × 1.43° visual angle), of which 155 were positive adjectives and 155 were negative adjectives. Each adjective consisted of two Chinese characters. The 210 of all words were presented in the encoding phase, and then all 310 words were presented in the recognition phase.

**FIGURE 1:**
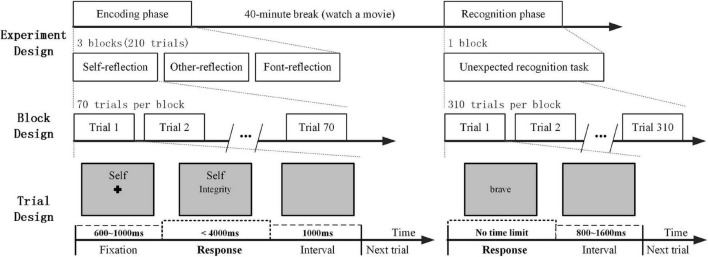
A schematic diagram of experimental design: the encoding phase and the recognition phase.

In the encoding phase, the subjects completed three blocks, and each block contained a task: self-reflection, other reflection, and font reflection. For the self-reflection task, the subjects judged whether this adjective described themselves or not. For the other-reflection task, the subjects judged whether this adjective described this familiar person or not. In addition, for the font-reflection task, the subjects judged whether the word was bold. In each task condition, there were 70 trials. There was a 600–1,000 ms time period with a cross (0.67° × 0.67° visual angle) shown at the beginning of each trial. Then, the subjects were asked to press the button within 4 s to respond with their left and right index fingers. The leftness and rightness of the responses were counterbalanced across the subjects. When the subject indicated their responses, the target word disappeared. The next trial was presented 1,000 ms later with the screen displaying gray and blank (Figure 1). During each trial, a small cue word (“self,” “other” or “font” in Chinese; 1.15° × 0.48° visual angle) remained in the upper part of the screen to remind the participants of the task in the block.

In the recognition phase, the subjects were asked to judge whether the words had appeared in the encoding phase by pressing the buttons. There was no time limit for the response. When the subjects made a choice, the word disappeared. Then, the next trial began after an interval of 800–1,600 ms. During a 40-min break between the encoding phase and the recognition phase, the subjects watched a movie that was unrelated to the experiment.

The stimulus display and behavioral data acquisition were conducted using E-Prime software (Version 2.0, Psychology Software Tools, Inc., Sharpsburg, PA, United States). The reaction time (RT) was measured in the encoding phase. During the recognition phase, the recognition score was assessed, which was defined as the difference from the proportion of hits and false alarms in each condition (Mu and Han, 2010; Philippi et al., 2012). In addition, the SRM bias score was defined by the differential recognition score between self and other reflection conditions for a comprehensive measurement (Zhao et al., 2016).

### Electroencephalogram Acquisition and Preprocessing

Sixty four channels of EEG data were recorded using a Brain Product system, which was connected to the recording computer (bandpass, 0.01–100 Hz; the sampling rate, 500 Hz/channel). The electrode locations conformed to the international 10–20 standard system. VEOG and HEOG were recorded by two pairs of electrodes, with one pair placed above and below the left eye and the other 10 mm from the left versus right orbital rim. Electrode impedance was kept below 5 kΩ throughout the experiment.

In this study, the data were preprocessed using EEGLAB (14.0.0b) in MATLAB R2014b. The average of the electrodes at the left and right mastoids was re-referenced, and the EEG data were filtered by a high-frequency cutoff of 45 Hz. EEGLAB provides an interactive graphic user interface, allowing users to flexibly and interactively process our EEG data using Independent Component Analysis (ICA). It performed the default ICA decomposition approach (runica). ICA can separate out certain types of artifacts. These include eye movements and eye blinks, temporal muscle activity, and line noise. If more than 30% of an independent component *z*-score kurtosis or skewness values, respectively, and outside ±2 of the distribution mean, we thought of the independent component as ocular or cardiac artifacts (Escudero et al., 2011; Dimitriadis et al., 2015). Then, a relatively artifact-free signal was reconstructed by the remaining artifacts. The average number of artifacts was 5.3 for the all conditions. The baseline was also corrected using a mean voltage activity of the 250-ms pre-stimulus period.

### Time-Frequency Power Analysis

To analyze the period and bands of EEG, the phase-locked spectral power was calculated to assess brain activity (Stothart and Kazanina, 2013). We analyzed the total frequency band responses with a complex Morlet wavelet using a wavelet toolbox in MATLAB. The wavelet family we used is defined as f0/sf = 7 (f0 ranging from 0.5 to 45 Hz in 0.5 Hz steps). The time-frequency representations of four bands of power were calculated for each subject. The TF power was shown from 0.5 to 45 Hz in a time window (250 ms pre-stimulus and 1,600 ms post-stimulus onset) for all electrodes. The topographies illustrated were mapped in three time windows (220–300, 420–500, and 600–1,600 ms) in the theta and alpha bands, which were used to analyze the distribution of activation energy throughout the brain.

In the study, we choose three time windows’ duration (220–300, 420–500, and 600–1,600 ms), and the three time windows were used to analyze the time-frequency activation energy and functional connectivity of the brain. Based on the results of time-frequency analysis and FC analysis in this paper, and combined with the results of previous studies, we found that the differences were more significant in these three time windows between normal people and patients with BD. More importantly, these three time periods represent key strategic steps in the task. The first time windows (220–300 ms): The P2 component is usually regarded as an attention-related biomarker at the early processing stage (Karayanidis and Michie, 1996; Chen et al., 2013). The second time windows (420–500 ms): The P3 component is the most noticeable marker of the self-referential effect (Chen et al., 2013), which is related to attentional resource allocation (Gray et al., 2004), and reflects making yes/no decisions (Chen et al., 2008). The third time windows (600–1,600 ms): the positive slow waves during 600–1,600 ms post-stimulus onset, reflect the integration of self-relevant information, a complex interaction of cognitive and emotional processing, following basic cognitive processing (Zhao et al., 2016).

### Functional Connectivity Estimation

Phase lag index was calculated between all pairs of EEG channels in each frequency band (theta, alpha, beta, and gamma bands) as a measure of functional connectivity. PLI measures the synchronization and can detect the extent of inter-trial phase variability between two signals in one given frequency across time (van Diessen et al., 2013; Wang et al., 2017; Zhang et al., 2018). The result of computing PLI for each time point was a 59 × 59 matrix in a frequency band, where 59 was the number of EEG channels after removing VEOG, HEOG, M1, and M2. This PLI provided the connection strength between electrodes. When the PLI is large; it showed that this non-zero phase locking is strong. These averaged PLI values in the whole brain were averaged for each task condition. The PLI values across sources for each time point reflected the dynamic network connectivity. To compare the differential PLI values of BD and NC, a two-sample *t*-test was performed at each time point.

### Feature Extraction Analysis of Dynamic Network Topologies

To characterize the network topology structure, we constructed a network (59 × 59) for each trial in a time window in the respective frequency band with GRETNA by using the time window identified in the above analysis (Wang et al., 2015). When we estimated the functional connectivity by PLI, for each time point, a functional connectivity matrix was obtained. We averaged fully weighted brain networks across 3 time windows, respectively: 220–300, 420–500, and 600–1,600 ms post-stimulus onset. We could get a sparse network by most common thresholding schemes, which maintain a specific ratio of the strongest edges. Then, the fully weighted graph was transformed into an adjacency graph, in which only significant edges are kept and the topological properties of the underlying network are better reflected (Dimitriadis et al., 2010, 2017a,b). We extracted the network according to the edge connection strength threshold so as to ensure that each node had at least one node connected to it. Then, we also analyzed the Clustering Coefficient of the sparse network to make sure that the network was connected. In order to avoid the contingency of the threshold selection, 11 thresholds (from 0.2 to 0.4, step value is 0.02) were selected for the sparse network separately. The results of the 11 times analyses were then averaged. For the analysis of brain networks, we calculated the brain network topology parameter of the path length (Lp), global efficiency (Eg), and degree (Deg). According to the TF power and the time courses of the averaged PLI, we still chose three time windows after the stimulus onset to characterize network connectivity in social cognitive processing. So, we used 2 (band) × 3 (task condition) × 3 (time window) × 3 (Lp, EG, and Deg) network topology parameters in the subsequent classifier analysis. In addition, the topography was mapped to observe the distribution of the degree values. For evaluating the local differences by group, the region of interest (ROI) was defined by repeated-measures ANOVAs on the Deg. The part of the group effect *p* < 0.05 was analyzed as the ROI. To further analyze the differences between groups, the independent-samples *t*-test was performed on ROI areas, respectively. A lucid flowchart on the analysis pipeline is provided in the supplementary material (Supplementary Figure 1).

### Classifier

To evaluate whether the parameters reached the advantage of automatic classification, we used a classifier based on machine learning methods. We used a support vector machine (SVM) classifier with a linear kernel, which was based on the LIBSVM library toolbox within MATLAB (Chang and Lin, 2011; Guo et al., 2012; Yang et al., 2018). To evaluate the classification performance, we used a cross-validation method. The 80% of the samples were randomly selected as a training set, and the remaining 20% were selected as a test set. For the test set, the prediction results were compared with the real labels, and then the accuracy (percentage of participants detected correctly) was calculated for one classification prediction. We mixed up all the samples after one training and testing. Then, we randomly selected test sets and test sets for the next training and testing. This process was repeated 100 times, and an overall accuracy was gotten by averaging the 100 prediction accuracy. The overall accuracy was calculated to quantify the classification performance, which reflected the predictive power of the parameter characteristic. Moreover, the area under the receiver operating characteristic curve (ROC) was also calculated to evaluate the classification performance. When the ROC value was increased, the classification performance was good, and the performance of the selected classification features was reliable. The classifier analysis was performed with all features of behavioral data, TF power, PLI, and network topology parameters. Then, the number of features reached the number of 94 per subject: 3 (a recognition score of three experimental conditions) + 1 (SRM bias scores) behavioral estimations; 2 (theta and alpha bands) × 3 (task conditions) × 3 (time windows) TF power estimations; 2 (bands) × 3 (task conditions) × 3 (time windows) PLI estimations; and 2 (bands) × 3 (task conditions) × 3 (time windows) × 3 (Lp, EG, and Deg) network topology parameters. The features were divided into three groups for execution based on the experimental conditions: self, other, font reflection condition and a combination of all conditions. Moreover, we performed multi-linear modeling of behavioral parameters with TF, PLI, and network metrics for three experimental conditions to further analyze importance of these metrics.

### Statistical Analysis

For the statistical analyses, we used SPSS version 20.0 software. For all the analyses in this study, the significance level was set at 0.05. The data in this study obey normal distribution.

#### Drug Factors

First, the characteristics between the two groups were compared by the Independent-Samples *t*-test. By using the equivalent dose conversion formula for antidepressants and antipsychotics, we performed an analysis of the correlation between drug factors and all experimental results for patients. There was no significant correlation between the two after Bonferroni correction (*p* > 0.05). Therefore, we ignored the influence of drug factors in the following analysis.

#### Repeated-Measures ANOVAs

Repeated-measures ANOVAs were conducted separately for the behavioral data and TF power, PLI values, and network topology parameters in each frequency band to examine the effects of group, experimental condition or time period. In our study, the repeated-measures ANOVAs analyzed all the factors and made pairwise comparisons in each *post hoc* analysis. SPSS software was used for ANOVA, and Bonferroni correction was carried out, so the output *P*-value was automatically multiplied by the comparison number K.

#### *t*-Tests

The independent sample *t*-tests were performed regarding differences from BD and NC. All statistical analyses were two tailed (α = 0.05). In the analysis of ROI, the independent-samples *t*-test also was adopted. The significant value was defined with 0.0042. Because there were 2 (band) × 3 (task) × 2 (ROI) conditions in this analysis, the result of *P*-value less than 0.0042 (α/K, conditions *K* = 12) was considered to be significant difference after correction.

## Results

### Behavioral Data Analysis

**FIGURE 2:**
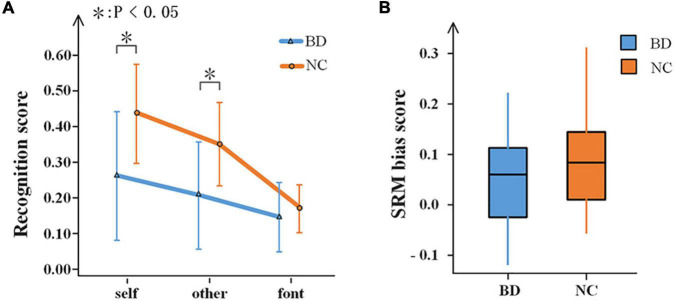
Behavioral results. **(A)** Recognition scores of BD and NC under the three experimental conditions. Error bars indicate the standard deviations. **(B)** A box-plot of the SRM bias scores in BD and NC. SRM, self-referential memory; BD, bipolar disorder; NC, normal control.

For the characteristics of the subjects, there was no significant difference between the two groups regarding age, education, or gender (*p* > 0.05). In the encoding phase, the RT of the target stimuli was evaluated. A 2-(group) ×3 (task condition) repeated-measures ANOVA performed on the RT revealed a significant effect of the group [*F*(1,40) = 25.68, *p* < 0.001]. BD was slower than NC in all referential conditions. For the recognition phase, 2-(group) × 3 (task condition) repeated-measures ANOVA was performed. The result is shown in Figure 2A. The main effect of the task condition was significant [*F*(2,39) = 36.169, *p* < 0.001], and all three tasks were significantly different from one another. There was a significant difference between BD and NC [*F*(1,40) = 10.466, *p* < 0.05]. There were significant interactions between the groups and the task conditions, *F*(2,39) = 5.828, *p* < 0.05. Thus, independent sample *t*-tests were conducted regarding the differences between BD and NC for each task condition. For self and other referential conditions, there were significant differences between BD and NC, *t* = −3.413, df = 40, *p* < 0.001, and *t* = −3.314, df = 40, *p* < 0.05, respectively. For font referential condition, there was no significant difference between BD and NC about the recognition scores (*p* = 0.325). Moreover, the SRM bias score was not significantly different between BD and NC (*p* = 0.268) according to independent sample *t*-tests analyses (Figure 2B).

### Phase-Locked Neural Activity

**FIGURE 3:**
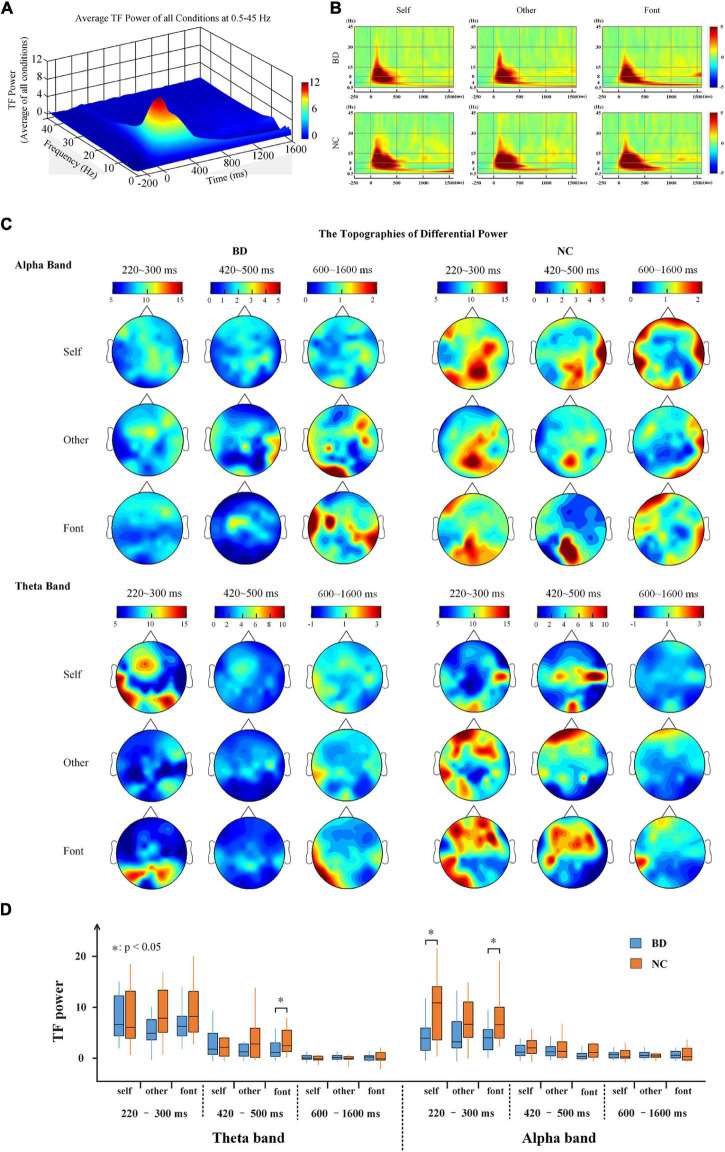
**(A)** The average TF power of all electrodes at 0.5–45 Hz from –250 to 1,600 ms after the onset is illustrated. **(B)** The TF power shows the phase-locked spectral power at 0.5–45 Hz from –250 to 1,600 ms in response to the three conditions at all electrodes. **(C)** The topographies illustrate the power distribution of the three time periods in the theta and alpha bands. **(D)** A box plot of the TF power for BD and NC under the three conditions in terms of the alpha and theta band activity levels. BD, bipolar disorder; NC, normal control; TF, time frequency.

The results showed that all conditions elicited an enhanced evoked power in the theta and alpha bands (Figures 3A,B). Moreover, the topographies of the brain (Figure 3C) showed that the whole brains of NC were more strongly activated than BD in the three time periods. For the theta and alpha bands, a 2-(group: BD, NC)- × 3 (task condition: self, other, and font referential) × 3 (time period: 220–300, 420–500, and 600–1,600 ms) repeated-measures ANOVA was performed. In the theta band, the main effect of time period was significant [*F*(2,80) = 37.915, *p* < 0.001], but no other main effects and interactions reached a significant level (*p* > 0.05). In the alpha band, the main effect of time period was also significant [*F*(2,80) = 47.212, *p* < 0.001]. Moreover, there was a significant difference between the groups [*F*(1,40) = 7.506, *p* < 0.05] and a significant interaction of time period × group [*F*(2,80) = 6.636, *p* < 0.05]. No other main effects or interactions reached a significant level (*p* > 0.05). Moreover, there were significant differences between BD and NC according to the independent sample *t*-tests analyses (Figure 3D).

### Scalp Topographies Along With the Functional Connections at the Electrodes Level

**FIGURE 4:**
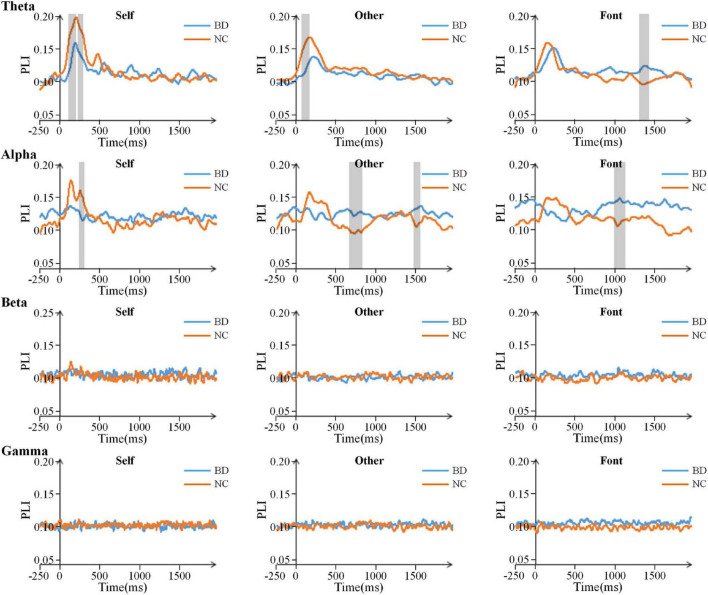
Time courses of the average PLI values for BD and NC in the four bands. The gray areas are the time windows in which there was a significant difference between BD and NC. BD, bipolar disorder; NC, normal control; PLI, phase lag index.

The PLI for all pairs of electrodes was calculated on the theta (4–8 Hz), alpha (8–13 Hz), beta (13–30 Hz), and gamma (30–45 Hz) frequency bands. After the stimulus onset, there was a significant increase in the value of PLI on the theta and alpha bands, especially in the early stage (Figure 4). The results showed that the PLI of NC was higher than that of BD in the early stage, but the PLI of BD increased in the later stage. This finding indicates that NC has faster brain responses than BD. However, there were only inconspicuous changes in network connectivity on the beta and gamma bands (Figure 4).

**FIGURE 5:**
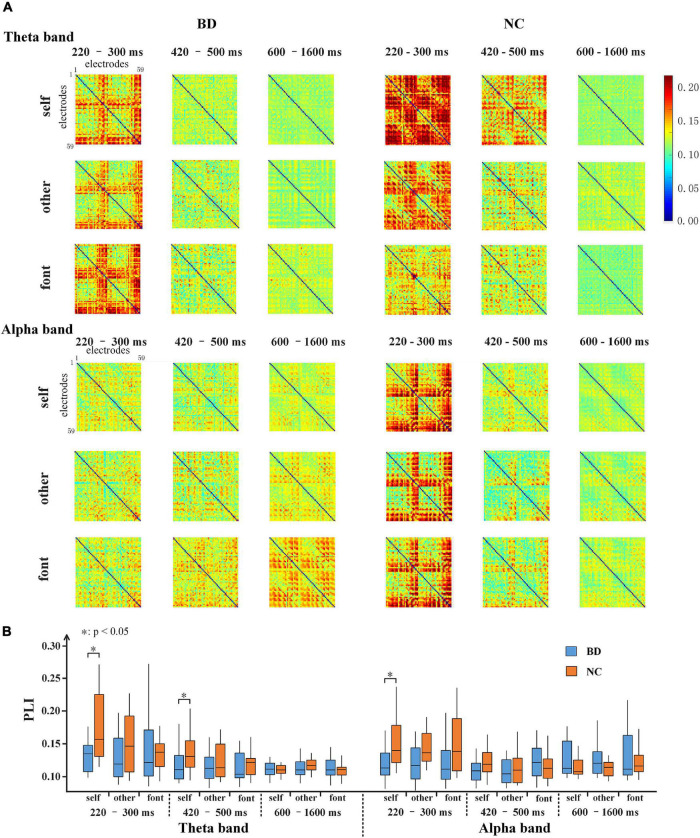
**(A)** 59 × 59 matrixes of average PLI within three time periods for BD and NC in three conditions in the theta and alpha bands. **(B)** A box figure of the average PLI values for BD and NC in the theta and alpha bands. BD, bipolar disorder; NC, normal control; PLI, phase lag index.

For the theta and alpha bands, the connection matrixes were averaged to acquire the averaged connection matrix of the three time periods (Figure 5A). The result of connection matrixes showed that the functional connectivity of NC was stronger than that of BD. A 2 (group) × 3 (task condition) × 3 (time period) repeated-measures ANOVA was performed. In the theta and alpha bands, the main effects of the time period were significant [*F*(2,80) = 17.730, *p* < 0.001 and *F*(2,80) = 4.618, *p* < 0.05, respectively], and there were significant interactions of the time period × group [*F*(2,80) = 5.826, *p* < 0.05 and *F*(2,80) = 7.344, *p* < 0.001, respectively]. Moreover, there was a significant interaction of time period × task condition [*F*(4,160) = 2.630, *p* < 0.05] in the theta band. No other main effects or interactions reached a significant level (*p* > 0.05). The significance differences mentioned above only existed under the self-referential condition (at 220–300 and 420–500 ms time windows on the theta band and 220–300 ms time window on the alpha band) by the independent sample *t*-tests analyses (Figure 5B).

### Network Topology Parameters

**FIGURE 6:**
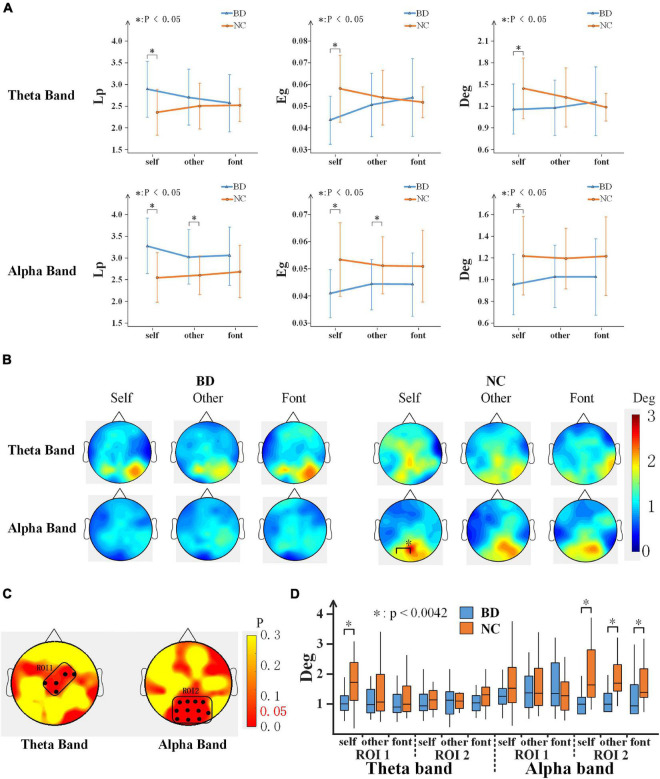
**(A)** Network topology parameter values of the three conditions for BD and NC in the theta and alpha bands at 220–300 ms. Error bars indicate the standard deviations. **(B)** The topography shows the distribution of the degree values for BD and NC in regard to the three conditions in terms of the theta and alpha band activity at 220–300 ms. **(C)** ROI (black dots) defined as the topography of ANOVA result. **(D)** The average Deg of two ROIs for BD and NC for all conditions. BD, bipolar disorder; NC, normal control; Lp, path length; Eg, global efficiency; Deg, degree; ROI, region of interest; ANOVA, analysis of variance.

To further analyze the differences in brain network connectivity between the groups, the path length (Lp), global efficiency (Eg), and degree (Deg) of the brain network were calculated. For each network topology parameter, a 2-(group) ×3 (task condition) repeated-measures ANOVA was performed. Significant differences were found only in the early stage (220–300 ms) (Figure 6A). On the theta band, there were significant interactions of the task condition × group for Deg [*F*(2,80) = 4.959, *p* < 0.05]. On the alpha band, the Lp value of BD was lower than that of NC [*F*(1,40) = 10.624, *p* < 0.05], the Eg [*F*(1,40) = 9.560, *p* < 0.05], and Deg [*F*(1,40) = 6.297, *p* < 0.05] were both larger than that of NC. According to the independent sample *t*-tests analyses between groups, there were significant differences for self and other referential conditions on the alpha band. However, there were significant differences for only the self-referential condition on the theta band (Figure 6A).

In addition, the Deg of each node was analyzed, as shown in Figure 6B, and the topographies of the brain were drawn based on the Deg of the 59 electrodes of the whole brain in a 220–300 ms time window after the stimulus onset. The results showed that NC demonstrated the activation of a larger area than BD. The large regions of the occipital lobe and parietal lobe showed significant activations. To analyze the difference in more detail between the groups, a region of interest (ROI) analysis was performed to the differences. The part of the group effect with significance (*p* < 0.05) was selected as the ROIs. According to the location, the two ROIs were divided (Figure 6C): ROI1 consists of C1, Cz, CPz, FC2, and FC4 electrodes, and ROI2 consists of P1, Pz, P2, P4, PO3, POz, PO4, PO8, O1, Oz, and O2 electrodes. The group effect on the Deg was significant between NC and BD in ROI1 [*F*(1,40) = 6.322, *p* < 0.05] and ROI2 [*F*(1,40) = 6.661, *p* < 0.05]. The Deg for NC was significantly higher than that for the BD in both ROIs. Further analysis shows that (Figure 6D), in the alpha band, the Deg of BD was significantly lower than NC in all experimental conditions in ROI2. In the theta band, however, the BD node degree was low only in the self-referential condition in ROI1.

### Classifiers

Table 1 depicts differences in the performance of the classifier model and ROC curves between BD and NC. Using the behavioral, TF power, PLI, and topology features, accuracies of 71.28, 65.86, 85.89, and 94.14% were achieved for the combination of all three referential conditions, respectively. This classification effect was sometimes higher than the three referential conditions alone. The average classifying accuracies of the three separate referential conditions for the four features were 61.68, 66.02, 73.96, and 87.73%, respectively. The results showed that the classification accuracy of the four features gradually increased, and the feature classification of the topology parameter worked best with the classifiers. It is worth noting that the classification accuracy of the PLI feature was as high as 86.97% in the self-referential condition.

**TABLE 1:** Accuracy and AUC of discriminating bipolar disorder from normal controls by the classifier based on machine learning methods.

Accuracy (AUC)	Behavioral	TF	PLI	Topology
All	71.28% (0.7301)	65.86% (0.6427)	85.89% (0.8956)	94.14% (0.9625)
Self	66.94% (0.6919)	60.50% (0.5907)	86.97% (0.9308)	85.72% (0.9120)
Other	63.11% (0.6389)	62.81% (0.6102)	71.83% (0.7011)	86.25% (0.8509)
Font	55.00% (0.5118)	74.75% (0.7500)	63.08% (0.6278)	91.22% (0.9310)

*The behavioral, TF power, PLI, and topology features were the input characteristic of the classifier for the combination or singleton of all three referential conditions, respectively. ROC, area under the receiver operating characteristic curve; TF, time frequency; PLI, phase lag index.*

The AUC scores were 0.7301, 0.6427, 0.8956, and 0.9625 for the behavioral, TF power, PLI, and topology features, respectively. The average AUC scores of three separate referential conditions were acquired using the four features and were 0.6142, 0.6503, 0.7532, and 0.8980, respectively. This result showed the same trend as the classifying accuracy. When combining three referential condition features, better AUC scores were achieved. From behavioral features to topology features, the AUC scores were higher as a whole.

Moreover, the multi-linear modeling indicated that the weight coefficients of the network metrics were the largest (self: 77.54%; other: 84.20%; font: 86.03%). The weight coefficients of the PLI were 22.39, 15.69, and 13.90% for three conditions, respectively. However, the weight coefficients of the TF power were less than 0.2% for all conditions.

## Discussion

We previously observed that self and other referential processing was impaired for patients with bipolar from the aspect of ERP characteristics (Zhao et al., 2016). We used brain functional connectivity to address the theoretical relationship between these brain functional network disturbances and the dysregulation of self-reference processing for BD. Our main findings were as follows: (1) patients with BD were slower to respond for self and other referential tasks and scored lower than normal; (2) for patients with BD, there was a lower TF energy of the whole brain, and the global connection strength of the EEG network was significantly reduced in the theta and alpha frequency bands, especially during self-referential processing; (3) the global topological organization in patients with BD was also transformed, such as an increased path length, decreased network clustering, and a lower nodal degree in self and other referential processing; and (4) from machine learning classification calculations, it was found that the brain connection had a good classification accuracy, especially the topological organization parameters of the network. Taken together, our findings support the hypothesis that the functional connection of the brain network is impaired in BD, especially in self-referential processing.

We analyzed TF power and the functional connection of the whole brain in three periods; the results showed that the value and the difference between BD and NC in the first period (220–300 ms) were greater than the other two periods. Then, this study was an exploratory analysis, and the selection of the frequency band for this analysis was *ex post facto* by our exploratory analysis. The results revealed that the TF power and brain functional connectivity of patients with BD became weaker compared with healthy controls in the theta and alpha bands; however, no similar phenomenon was found in the beta and gamma bands. Therefore, in this paper, we analyzed and discussed the results in detail of these 220–300 ms time windows of the theta and alpha frequency bands. Because N250 and P300 are regarded as indicators of familiarity and allocation of attention resources, they are the main ERP components related to self-referential processing (Ninomiya et al., 1998; Northoff et al., 2006). Many studies have shown that the self-referential effect was likely to produce larger than P300 amplitudes, which suggests that self-referential stimuli recruit a large amount of attentional and cognitive resources (Gray et al., 2004; Chen et al., 2008; Zhao et al., 2016). Moreover, the studies reported a decreased power in the alpha band (Basar et al., 2012) and the theta band (Howells et al., 2012) in patients with BD. The other EEG study also showed a decline in alpha power in the rest state for patients with BD (Kim et al., 2013). Decreases in synchronization in the alpha band and connections at fronto-central and centro-parietal were found in BD (Kim et al., 2013). Therefore, the findings suggest abnormal theta and alpha band activity during self- and other referential processing in the 220–300 ms range after stimulation for BD.

Regarding the functional connectivity of the whole brain, we inferred that the brain of BD was deficit in self- and other referential processing. Previous studies have provided a great deal of evidence regarding the impairment of brain functional connectivity in BD. An analysis showed that there were potential functional consequences in brain networks in BD (Kam et al., 2013; Handayani et al., 2017; Jeganathan et al., 2018). From brain network analyses, the impaired brain region integration was revealed in BD. Then, our findings also showed more activated connection areas of the brain in NC than in BD during self and other referential processing. This indicates that self-cognition of BD was blocked in the process of brain interval information transmission and integration, suggesting functional connectivity alterations may be an important feature of the disorder (Versace et al., 2010).

The results of the topological structure further verify and explain this point of view. This study found several altered network parameters in patients with BD. Specifically, the Eg and Deg were significantly decreased in the alpha and theta bands, while the characteristic Lp was increased. Similarly, the common phenomenon for several diseases and modalities was an increase in path length, which showed an inefficient brain network in patients with brain disorders (Stam et al., 2009; Lo et al., 2010; van Wijk et al., 2010). These results were similar to a recent network study in BD, which found a decreased clustering coefficient, decreased Eg and increased Lp (Leow et al., 2013). Using rest state fMRI and EEG, increased Lp and decreased network efficiency were found in major depressive disorder (Leistedt et al., 2009; Zhang et al., 2011). This brain network defect was also manifested in social cognitive processing. In detail, BD had defects in both self- and other referential processing within the alpha bands; however, in the theta band, the defect of BD was only reflected in self-referential processing. Moreover, PLI intensity connections have been found to be associated with cognitive deficits in the body, although not significantly. A previous study found that the dorsolateral prefrontal cortex was activated in self-referential processing rather than other referential processing (Kelley et al., 2002). Gray reported that the amplitude of P300 induced by self-related stimulus was larger than that of non-self (Gray et al., 2004). Self-evaluation is a kind of self-referential processing with a complex structure, and we believed that there were brain network differences from self and other referential processing. To further analyze the impaired connection of BD, the ROI analysis was implemented. As the above results, the Deg was significantly lower in the BD than in NC, especially in the occipital lobe and right parietal lobe areas. A study showed the decreases connections at fronto-central and centro-parietal were found in BD (Kim et al., 2013). The connection abnormality of parietal lobe areas in BD was also found (Jeganathan et al., 2018). Taken together, the patients were unable to integrate and convey information, resulting in a loss of efficiency and ability in social cognitive processing, especially in self-referential processing.

Combining the behavioral performance, the patients with BD had a significantly slower reaction and lower recognition scores in the self and other referential tasks. This showed that both self-referential and other referential processing were disrupted for BD, which was similar to the results of a previously published paper (Zhao et al., 2016). However, the SRM bias score was not significantly different between the healthy subjects and patients with BD. The results were less efficient in the cognitive process but had little effect on the cognitive outcome. Although the brain function is aberrant for BD, we think it may be a manifestation of the compensatory mechanism that compensates for some of the cognitive deficits so that there is no difference in behavioral representation self- and other referential processing.

In the present study, we demonstrated that discriminating between BD and NC with high accuracy and AUC values can be possible by using an SVM classification. A maximum classification accuracy of 94.14% was obtained when topology features of all conditions were selected. The classification accuracy obtained in this study was considerably precise compared with those of previous research findings (Librenza-Garcia et al., 2017; Maggioni et al., 2017). Moreover, the topological parameters and PLI still had good classification characteristics under a single condition. Notably, the accuracy was also very high for the topological parameters of the font-referential condition. It is hard to provide a proper reason as to why a particular classifier shows a better performance than others since classification accuracies of different classifiers highly depend on the given data sets. However, the high identification accuracies demonstrated that both PLI and topology could depict the functional alterations of brain regions in BD. This procedure was efficient and robust, indicating that PLI and topology features could be a potential index to monitor disease progression. The results of the multi-linear modeling analysis also confirmed this inference.

Our study has some limitations that can be compensated for in future studies. All the patients with BD were taking psychotropic medications. Although the results showed no significant correlation between drug factors and all experimental results for patients with BD, we did not analyze the drug’s effect on the experiment in depth. To avoid the influence of drug factors, the current modes should be repeated in non-medicated samples in future studies by recruiting patients with first episode BD, who were not taking antidepressants and antipsychotics. In addition, the patient groups were hospitalized inpatients; hence, it is unclear whether the same findings would be observed in outpatients, which should be addressed in the future. Moreover, only 59 channels were used in this study. If the EEG source imaging or brain regions are divided, the location may be inaccurate due to the insufficient number of electrodes, which cannot guarantee the reliability of the statistical results. In the future, we can repeat the experiment with more channels EEG, such as 128 channels. Then, by focusing on the ROI brain region, the abnormal brain region and the connections in patients with BD were further studied.

In the present study, we found that behavioral assessment results were poor and TF power was weak over the whole brain of BD from the social cognitive performance in 220–300 ms after stimulation. Mainly, this study represented the brain network analyses in BD. The results revealed weaker brain connections in BD. The analyses of dynamic network topologies further revealed that BD showed a longer path length, a lower global efficiency, and a lower node degree compared with NC on a global level, especially in self-referential processing. These results suggested that patients with BD were unable to integrate whole brain resources into the self-referential process, which led to a loss of efficiency and ability. In addition, our results of the SVM classification, which set useful strategies for future research, demonstrated functional alterations of brain regions in BD.

## Data Availability Statement

The raw data supporting the conclusions of this article will be made available by the authors, without undue reservation.

## Ethics Statement

The studies involving human participants were reviewed and approved by The Research Ethics Committee of the Beijing HuiLongGuan Hospital and Beijing HuiLongGuan Hospital. The patients/participants provided their written informed consent to participate in this study.

## Author Contributions

JZ analyzed and interpreted the data and wrote the manuscript. ZS and DS conceived and designed the experiments. TL, ST, SF, and CD performed the experiments and revised the manuscript. LW, JW, and ML approved the final version. All authors contributed to the article and approved the submitted version.

## Conflict of Interest

The authors declare that the research was conducted in the absence of any commercial or financial relationships that could be construed as a potential conflict of interest.

## Publisher’s Note

All claims expressed in this article are solely those of the authors and do not necessarily represent those of their affiliated organizations, or those of the publisher, the editors and the reviewers. Any product that may be evaluated in this article, or claim that may be made by its manufacturer, is not guaranteed or endorsed by the publisher.
