# Supplementary material for: Impaired Self-Referential Cognitive Processing in Bipolar Disorder: A Functional Connectivity Analysis
Source: Front Aging Neurosci. 2022 Feb 7;14:754600. doi: 10.3389/fnagi.2022.754600 (PMC8859154; doi:10.3389/fnagi.2022.754600)
Supplement: Supplementary file 1 [file Data_Sheet_1.pdf]

Supplementary Material

1 Supplementary Figures

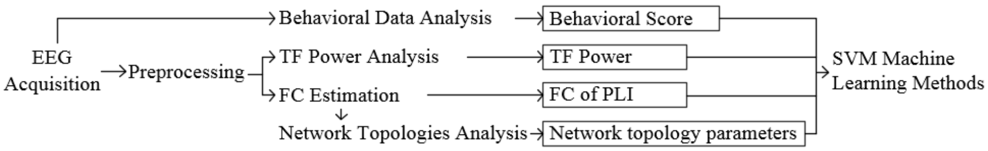

Supplementary Figure 1. The flowchart on the analysis pipeline and sequence.

2 Supplementary Tables

Supplementary Table 1. Demographic and clinical data for patient and control groups.

Characteristics	Patients (BD)	Controls (NC)
number	23	19
Age (years)	34.3 ± 12.0	32.6 ± 8.2
Education time (years)	12.60 ± 3.22	13.50 ± 2.71
Gender, male/female	12/11	11/8
Handedness, right/left	23/0	19/0
Age at onset (years)	23.80 ± 8.30	
Duration of illness (years)	10.50 ± 9.05	
CGI-S	5.13 ± 1.25	
Depression (HAMD)	6.20 ± 8.61	
Mania (YMRS)	18.20 ± 10.53	
